# Hierarchical Quantum Embedding by Machine Learning for Large Molecular Assemblies

**DOI:** 10.1021/acs.jctc.5c00389

**Published:** 2025-07-29

**Authors:** Moritz Bensberg, Marco Eckhoff, Raphael T. Husistein, Matthew S. Teynor, Valentina Sora, William Bro-Jørgensen, F. Emil Thomasen, Anders Krogh, Kresten Lindorff-Larsen, Gemma C. Solomon, Thomas Weymuth, Markus Reiher

**Affiliations:** † 27219 ETH Zurich Department of Chemistry and Applied Biosciences, Vladimir-Prelog-Weg 2, 8093 Zurich, Switzerland; ‡ 31064 University of Copenhagen Department of Chemistry and Nano-Science Center, Universitetsparken 5, DK-2100 Copenhagen Ø, Denmark; § NNF Quantum Computing Programme, Niels Bohr Institute, University of Copenhagen, Blegdamsvej 17, DK-2100 Copenhagen Ø, Denmark; ∥ 6756 University of Copenhagen Department of Computer Science, Universitetsparken 1, DK-2100 Copenhagen Ø, Denmark; ⊥ University of Copenhagen, Department of Biology, Linderstrøm-Lang Centre for Protein Science, Ole Maaløes Vej 5, DK-2200 Copenhagen N, Denmark; # University of Copenhagen, Center for Health Data Science, Department of Public Health, Øster Farimagsgade 5, DK-1353 Copenhagen Ø, Denmark

## Abstract

We present a quantum-in-quantum embedding strategy coupled to machine learning potentials to improve on the accuracy of quantum-classical hybrid models for the description of large molecules. In such hybrid models, relevant structural regions (such as those around reaction centers or pockets for binding of host molecules) can be described by a quantum model that is then embedded into a classical molecular-mechanics environment. However, this quantum region may become so large that only approximate electronic structure models are applicable. To then restore accuracy in the quantum description, we here introduce the concept of quantum cores within the quantum region that are amenable to accurate electronic structure models due to their limited size. Huzinaga-type projection-based embedding, for example, can deliver accurate electronic energies obtained with advanced electronic structure methods. The resulting total electronic energies are then fed into a transfer learning approach that efficiently exploits the higher-accuracy data to improve on a machine learning potential obtained for the original quantum-classical hybrid approach. We explore the potential of this approach in the context of a well-studied protein–ligand complex for which we calculate the free energy of binding using alchemical free energy and nonequilibrium switching simulations.

## Graphical abstract

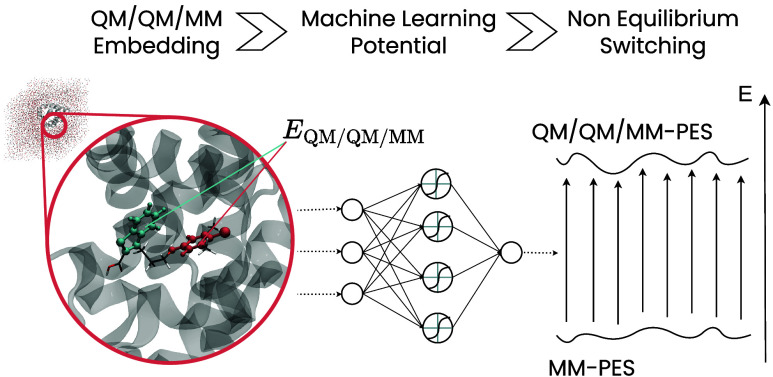

## 1 Introduction

Hybrid models for large macromolecules can leverage a structural decomposition into a scaffold (spectator) part, which can be described by classical molecular mechanics (MM) force fields, and into a smaller quantum region described by a quantum mechanical (QM) method. Such a decomposition is routinely applied to describe local phenomena, such as catalytic chemical reactions mediated by metal ions in metalloenzymes or the binding of drug molecules to proteins. However, to obtain reliable results that do not suffer from artifacts caused by the MM description of the scaffold, large QM regions are required^1–4^ to which only approximate electronic structure methods (such as density functional theory (DFT) or even fully semiempirical quantum chemical approaches) can be applied. More accurate electronic structure models are hardly applicable because of their steep scaling of computational costs with system size. The accuracy of DFT for the QM region may be enhanced by quantum-in-quantum (QM/QM) embedding approaches. For instance, Morokuma’s ONIOM model^5,6^ is a subtractive approach allowing the combination of arbitrary QM and MM methods into multilayer embedding schemes. In ONIOM, the contribution of the embedded regions is estimated as the difference between the high level (HL) and low level (LL) energies for the region. This scheme makes the implementation of ONIOM easy. However, it also restricts how the embedding regions can be polarized by one another. Alternatively, additive approaches such as density-matrix embedding theory,^7^ subsystem density functional theory,^8,9^ Carter’s wave function-in-DFT embedding,^10^ bootstrap embedding^11^ or projection-based embedding^12,13^ allow for a direct coupling between the low-level environment and the high-level wave function through DFT or bath orbitals, providing a potentially more accurate description of the interaction.

Recently, several studies investigated projection-based embedding in the context of QM/QM/MM approaches to study the reactivity in protein complexes (see refs 14–17 for examples). However, more general biochemical problems such as the calculation of binding free energies have not been targeted with modern DFT-based QM/QM embedding approaches. Here, we describe such a general approach, where we exploit a machine learning potential (MLP) representation to mediate between the different types of data to be combined in a single hybrid model. First, we rely on our previous work^18^ where we have shown how to construct an MLP for a full QM/MM model and how to exploit this MLP representation in a free-energy sampling approach. Second, we combine this approach with a QM/QM refinement step for accuracy enhancement that is mediated by transfer learning toward a refined MLP representation.

In our QM/QM/MM embedding approach, selectively refining an available quantum description in a QM/MM hybrid model is achieved by defining one or more quantum cores within the quantum region. A Huzinaga-type projection-based embedding approach^19^ allows us to improve the quantum description within the quantum cores. This information is then exploited in a transfer-learning approach that replaces the explicit quantum energies in the QM/QM/MM model with a machine-learning-in-MM (ML/MM) energy expression.

Although our approach is general and will work for any QM/MM modeling approach toward physicochemical properties, we focus on binding free energies for protein–ligand complexes, which characterize the affinity and specificity of drug-to-target binding, making them invaluable information for drug discovery and for understanding molecular recognition in general. We demonstrate our QM/QM/MM and transfer learning strategy for the prediction of the binding of an inhibitor (19G) to the myeloid cell leukemia 1 (MCL1) protein^20^ (protein data bank entry 4HW3).

This work is structured as follows: First, we introduce our workflow, the QM/QM/MM, and the transfer machine learning approach in Section 2. We then discuss the effect of QM/QM/MM compared to traditional QM/MM embedding for electronic energies in Section 4.1, characterize the accuracy of our MLP in Section 4.2, and also discuss the effect of energy derivatives on the ML training procedure in Section 4.3. Finally, we present our QM/QM/MM estimate for the binding free energy of 19G to MCL1 in Section 4.4.

## 2 Theory of Machine Learning Assisted Multilevel Embedding

### 2.1 Binding Free Energies from Machine Learning Abstracted First-Level Embedding

We have shown in previous work^18^ that the free energy of binding of a ligand to a protein can be calculated with an ML/MM approach by correcting the end states of classical MM-based alchemical free energy calculations^21,22^ following the thermodynamic cycle shown in Figure 1(a). In this approach,^18^ we trained an ML potential to reproduce a QM/MM potential energy surface accurately. As a demonstration of the fidelity of the emerging potential energy hypersurface, we performed nonequilibrium switching to calculate the free energy difference of switching from the MM potential energy surface to the ML/MM potential energy surface. The end states for which a correction was required were the protein–ligand complex and the solvated ligand. For details on this methodology, we refer the reader to ref 18.

**Figure 1:**
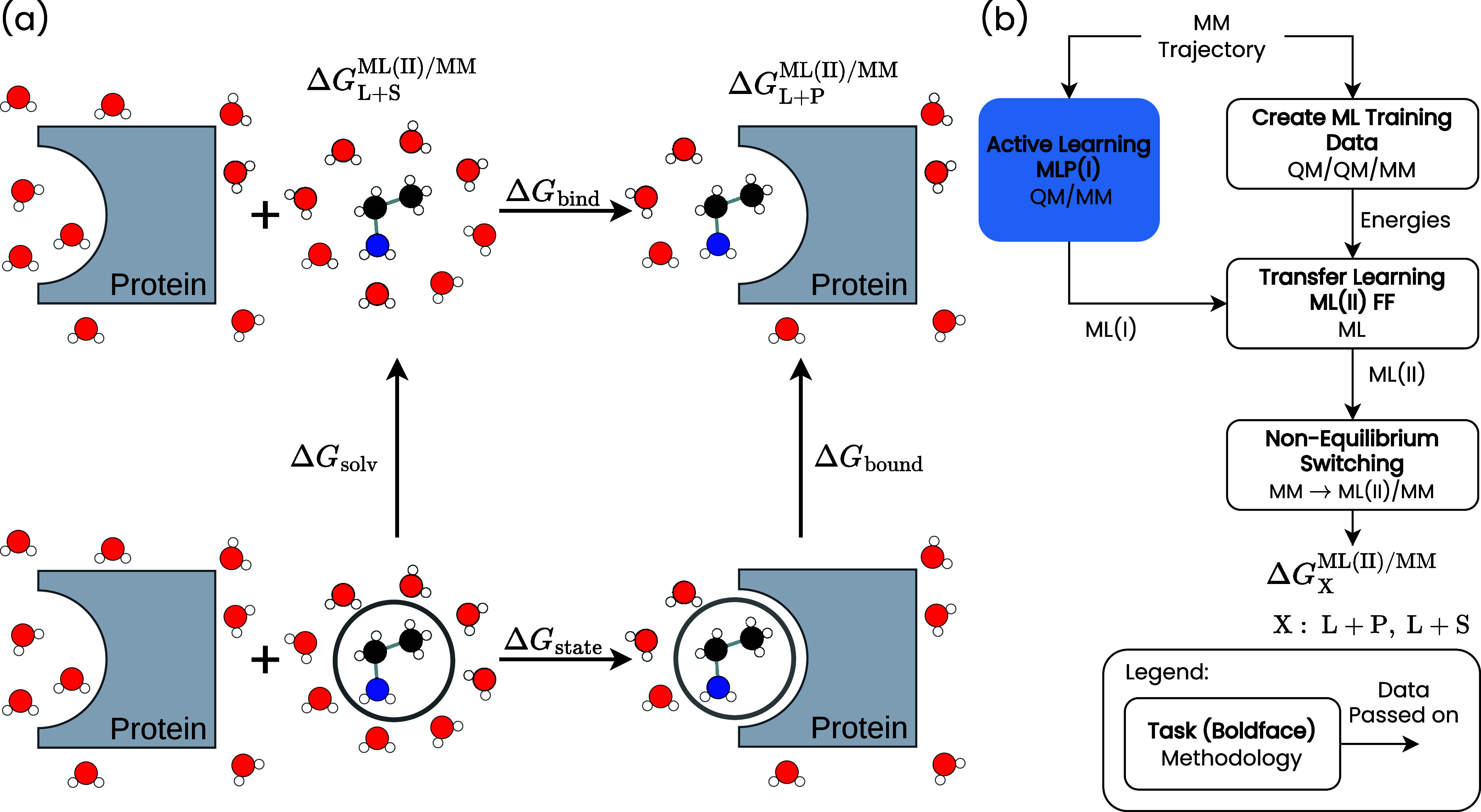
(a) Thermodynamic cycle to calculate the binding free energy of the ligand to the protein Δ*G*_bind_. The black circles indicate that there is no interaction between the ligand and the solvent or the ligand and the solvated protein. (b) Transfer learning strategy as required for the nonequilibrium switching from the MM to the ML­(II)/MM force fields (FFs).

Compared to our previous work,^18^ we now improve on the MLPs by adopting more accurate quantum energies through transfer learning, as shown in Figure 1(b). We here reoptimize the parameters of the MLPs from ref 18 [from here on denoted as ML­(I)] by considering data from correlated quantum chemical methods obtained for quantum cores embedded in the original quantum region. We extracted snapshots from the MM trajectories and the structures generated during active learning used in the original workflow^18^ and calculated QM/QM/MM energies for them. With these energies, we trained a revised MLP, denoted ML­(II), and performed nonequilibrium switching from the MM potential energy surface to the ML­(II)/MM potential energy surface for the end states, that is, for the protein–ligand complex and the solvated ligand. In this work, we relied on energies only to train the ML­(II)/MM potential because energy gradients have not been implemented for our QM/QM model yet. Including energy gradients in the training generally reduces the required training data because the gradients provide significantly more information on the potential energy surface than the energy data alone.^23^ While we address and assess this issue later in this paper, we note that the development of a rigorously defined QM-in-QM embedding gradient is not straightforward in our framework and must therefore be deferred to future work.

Furthermore, we performed switching simulations starting from the MM surface because we require an equilibrium ensemble from which to start the switches. Therefore, we avoid equilibrating the protein–ligand complex and the solvated ligand with the ML­(I)/MM machine learning potential. In principle, this would have been another option. However, equilibration with ML­(I)/MM would require significant computational time because of the slow simulation speed of ML potentials compared to MM force fields.

### 2.2 Quantum Core Selection

To demonstrate the effect of our QM/QM/MM embedding strategy on the free energy of binding, we manually selected two quantum cores from the QM region generated in ref 18 for the MCL1–19G complex. The full system, including solvent and protein environment, is shown in Figure 2(a), where we highlighted the QM region and color-coded the two quantum cores. Furthermore, we show the ligand’s Lewis structure with the quantum cores in Figure 2(b). The same quantum core definition was used for all end states.

**Figure 2:**
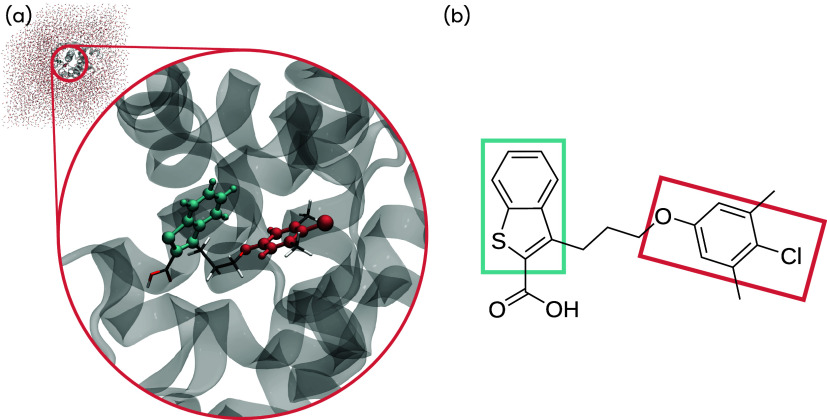
(a) Illustration of the protein–ligand complex. The QM region is drawn as a stick model, and the quantum cores are represented as balls and sticks. The two quantum cores are highlighted by color. (b) Lewis structure of the ligand 19G. The boxes highlight the quantum cores.

In principle, the quantum cores may be selected to include areas of the system contributing strongly to the interaction between protein and ligand since this interaction is effectively sampled during alchemical free energy simulation. However, as we will show in this paper, improving the QM region at the ligand is advantageous for increasing the overall accuracy of the QM/QM/MM calculation. Hence, since we adopt the QM region from ref 18, which comprises the ligand atoms, the quantum core considered in this work is also restricted to the ligand atoms. However, we note that the quantum core could, in principle, be placed anywhere within the large QM region of a QM/MM model.

The quantum cores are the separated aromatic moieties of the ligand. We selected these quantum cores because they are important for the ligand−protein interaction. The aromatic systems are arranged in a T-shaped structure with aromatic residues in the protein environment, leading to a favorable noncovalent interaction between ligand and protein due to the quadrupole moment of the aromatic systems. Note that the quantum cores may be chosen to be larger and include also close atoms of the protein. With such an extended QM region, the aromatic residues of the protein could also be assigned to the quantum cores to increase the accuracy at which the interaction between the aromatic systems is described.

Furthermore, the quantum core selection can be automated by identifying parts of the electronic structure in the QM region that contribute strongly to the interaction between the ligand and the environment. For instance, an energy decomposition analysis^24,25^ applying computationally efficient DFT models could be used to highlight which fragments contribute strongly to the ligand-protein interaction. Alternatively, the orbitals of the QM region could be analyzed automatically^26,27^ to identify the part of the electronic structure in the QM region that contributes strongly to the variation in the relative energies and, therefore, requires the most accurate description.

We also note that it is, in principle, also possible to sample the large quantum region fully with smaller quantum cores by partitioning the system so that all atoms of the quantum region are assigned to some quantum core.

### 2.3 Huzinaga-Type Projection-Based Embedding

In our QM/QM/MM embedding approach, we aim to describe the quantum cores by an accurate (in general, that will be a correlated) wave function method and the remaining QM region with a more approximate electronic structure model such as DFT. For this approach, we require a suitable embedding technique and selected Huzinaga-type projection embedding for this purpose.^19^ However, other options—such as bootstrap embedding^11,28^ –exist and could be selected instead.

We define some notation for the sake of clarity: (i) Since Huzinaga-type embedding introduces a local correction to the electronic structure, we will denote our methodology as “QM­(HL)/QM­(LL)/MM” in the following. Here, “HL” and “LL” denote high level and low level, respectively, and refer to the expected accuracy of the underlying electronic structure model. (ii) We will refer to all atoms associated with QM­(HL) and QM­(LL) as the “QM region” (they therefore comprise all atoms that are captured by any quantum description). All remaining atoms belong to the MM region. (iii) When referring to the electronic structure, we will either refer to orbital sets associated with QM­(HL) or QM­(LL) separately, or we will refer to their union HL ∪ LL if the electronic structure described by all orbitals associated with the QM region is indicated. Note that we will be using orbital sets instead of terms like fragments or subsystems because the individual nuclei associated with a fragment or subsystem have no relevance for the formal definitions in Huzinaga-type QM/QM embedding.

We write the total energy of our system *E*_QM/QM/MM_ as the sum of the energy of the molecular mechanics model *E*_MM_, the energy of the capped QM region *E*_QM_, the electrostatic interaction energy *E*_elec_^int^, and the nonelectrostatic interaction energy *E*_LJ_^int^ of the QM and MM region

1 $$E_{\mathrm{QM}/\mathrm{QM}/\mathrm{MM}}=E_{\mathrm{QM}}+E_{\mathrm{MM}}+E_{\mathrm{elec}}^{\mathrm{int}}+E_{\mathrm{LJ}}^{\mathrm{int}}$$

In contrast to ref 18, we now increase the accuracy of *E*_QM_ through Huzinaga-type projection-based embedding, which allows us to apply correlated quantum chemical methods to increase the accuracy of the description of the quantum cores.

For Huzinaga-type projection-based embedding, we first optimize and localize the Kohn–Sham orbitals (HL ∪ LL) for the QM region. We then partition the full set of occupied Kohn–Sham orbitals into sets for each quantum core and a set of remaining orbitals; these are the environment orbitals, also denoted the LL orbitals. Note that any orbital localization or partitioning approach, such as intrinsic bond orbitals^29^ or Pipek–Mezey orbitals,^30^ can be applied in this step.

We write the total QM energy for our embedding approach as a sum of all energies for these quantum cores *E*_*I*_^QC^, the Kohn–Sham energy of the LL orbitals *E* ^LL^, and the rest energy *E* ^rest^, correcting for any remaining energy contributions

2 $$E_{\mathrm{QM}}=\sum_{I}E_{I}^{\mathrm{QC}}+E^{\mathrm{LL}}+E^{\mathrm{rest}}$$

The energy of the LL orbitals is calculated from a typical Kohn–Sham DFT expression

3 $$E^{\mathrm{LL}}=\sum_{i\in \mathrm{occ}\left(\mathrm{LL}\right)}\left\langle i\right|\mathrm{t̂}+{\mathrm{v̂}}_{\mathrm{ext}}\left|i\right\rangle +\int \int \frac{\rho_{\mathrm{LL}}\left(r^{\prime }\right)\rho_{\mathrm{LL}}\left(r\right)}{\left|r^{\prime }-r\right|}dr^{\prime }dr+E_{\mathrm{xc}}\left[\rho_{\mathrm{LL}}\right]$$

where the sum runs over all LL occupied orbitals, *t̂* is the kinetic energy operator, *v̂*_ext_ collects the Coulomb potential energy operators of the QM nuclei and those of the MM embedding charges, ρ_LL_ is the electron density of the low-level orbital set, and *E*_xc_ is the exchange–correlation functional evaluated for the LL-electron density. We note that the LL-electron density will feature holes where the quantum-core densities have been carved out owing to the embedding procedure. The energy of each quantum core is given as

4 $$E_{I}^{\mathrm{QC}}=\left\langle \Psi_{I}\left|{Ĥ}_{I}\right|\Psi_{I}\right\rangle$$

where Ψ_*I*_ is the wave function of the quantum core *I* defined in terms of its occupied orbital space and the full Kohn–Sham virtual orbital space of the (complete) QM region. The Hamiltonian operator *Ĥ*_*I*_ is defined in second quantization as

5 $${Ĥ}_{I}=\sum_{\mathrm{pq}}h_{\mathrm{pq}}^{I}a_{p}^{†}a_{q}+\sum_{\mathrm{pqrs}}g_{\mathrm{pqrs}}a_{p}^{†}a_{q}a_{r}^{†}a_{s}$$

where *a*_*p*_^†^ and *a*_*p*_ denote the creation and annihilation operators for *p*, respectively. The integral *g*_*pqrs*_ is the two-electron interaction integral, and *h*_*pq*_^*I*^ is the one particle integral. Only the integral *h*_*pq*_^*I*^ = ⟨*p*|*ĥ* ^*I*^|*q*⟩ is modified in our embedding approach compared to a nonembedded calculation. The operator *ĥ* ^*I*^ is defined as

6 $${ĥ}^{I}=\mathrm{t̂}+{\mathrm{v̂}}_{\mathrm{ext}}+{\mathrm{v̂}}_{\mathrm{xc}}^{\mathrm{nadd},I}+{\mathrm{v̂}}_{C}^{I}+{\mathrm{p̂}}^{I}$$

where *v̂*_xc_^nadd,*I*^ is the nonadditive exchange–correlation potential^8^

7 $${\mathrm{v̂}}_{\mathrm{xc}}^{\mathrm{nadd},I}\left(r\right)={\frac{\delta E_{\mathrm{xc}}\left[\rho \left(r\right)\right]}{\delta \rho \left(r\right)}|}_{\rho =\rho_{\mathrm{tot}}}-{\frac{\delta E_{\mathrm{xc}}\left[\rho \left(r\right)\right]}{\delta \rho \left(r\right)}|}_{\rho =\rho_{I}}$$

ρ_*I*_ denotes the density of the quantum core *I* and ρ_tot_ denotes the total density of the QM region (ρ_tot_ = ∑_*I*∈HL_ρ_*I*_ + ρ_LL_). The Coulomb potential created for the *I*th-quantum-core electrons by electrons in all other quantum cores *J* and the LL orbitals is given by *v̂*_C_^*I*^

$${\mathrm{v̂}}_{C}^{I}=\underset{J\neq I}{\sum }\int \frac{\rho_{J}\left(r^{\prime }\right)}{\left|r-r^{\prime }\right|}dr^{\prime }+\int \frac{\rho_{\mathrm{LL}}\left(r^{\prime }\right)}{\left|r-r^{\prime }\right|}dr^{\prime }$$ 8

The operator *p̂* ^*I*^ is a projection operator ensuring orthogonality between orbital sets.^19^ It is defined as

9 $${\mathrm{p̂}}^{I}=-\sum_{\begin{array}{l} J\neq I\\ J\in \mathrm{HL}\cup \mathrm{LL} \end{array}}{\mathrm{P̂}}_{J}\left({\mathrm{f̂}}^{I}-\epsilon_{\mathrm{shift}}\right)-\left({\mathrm{f̂}}^{I}-\epsilon_{\mathrm{shift}}\right)\sum_{\begin{array}{l} J\neq I\\ J\in \mathrm{HL}\cup \mathrm{LL} \end{array}}{\mathrm{P̂}}_{J}$$

where *P̂*_*J*_ = ∑_*i*∈occ_|*i*⟩⟨*i*| is the projection operator projecting onto the occupied orbitals of the environment orbital set (quantum core or DFT environment) *J*, *f̂* ^*I*^ is the embedded Fock operator of quantum core *I*

10 $${\mathrm{f̂}}^{I}={\mathrm{f̂}}_{\mathrm{HF}}^{I}+{\mathrm{v̂}}_{\mathrm{xc}}^{\mathrm{nadd},I}+{\mathrm{v̂}}_{C}^{I}$$

and ϵ_shift_ is a constant positive shift.^31,32^ The operator *f̂*_HF_^*I*^ is the Hartree–Fock Fock operator of quantum core *I*. Note that the shift ϵ_shift_ prevents a shift of environment orbitals to lower energies (producing a violation of the Aufbau principle) but does not affect the physics of the embedding procedure.

The remaining energy contributions and corrections are collected in *E* ^rest^

11 $$E^{\mathrm{rest}}=E_{\mathrm{xc}}\left[\rho_{\mathrm{tot}}\left(r\right)\right]-\sum_{I\in \mathrm{HL}\cup \mathrm{LL}}E_{\mathrm{xc}}\left[\rho_{I}\left(r\right)\right]-\sum_{I\in \mathrm{HL}}\left\langle \Psi_{I}\left|{\mathrm{v̂}}_{\mathrm{xc}}^{\mathrm{nadd},I}\right|\Psi_{I}\right\rangle -\sum_{\begin{array}{l} I<J\\ I,J\in \mathrm{HL} \end{array}}\int \int \frac{\rho_{I}\left(r^{\prime }\right)\rho_{J}\left(r\right)}{\left|r^{\prime }-r\right|}dr^{\prime }dr$$

The first two terms are the nonadditive exchange–correlation energy contributions used to describe the exchange–correlation interaction in Huzinaga-type DFT embedding. We corrected this energy by subtracting the contribution of *v̂*_xc_^nadd,*I*^ from eq 6 because the expectation value of the exchange–correlation potential is, in general, not identical to the exchange–correlation functional *E*_xc_. Therefore, we must evaluate the nonadditive contribution of *E*_xc_ explicitly (first and second term on the r.h.s.). Furthermore, the Coulomb interaction between the quantum cores would be considered twice through the operator *v̂*_C_^*I*^, which is why the Coulomb interaction between electrons in different quantum cores must be subtracted (fourth term on the r.h.s.) to avoid double counting. Note that in practice, only the Kohn–Sham DFT densities of the orbital sets are used to evaluate the rest term *E* ^rest^ since densities or density matrices for the correlated wave functions of the quantum cores may not always be available.

Moreover, we avoid the embedded self-consistent field procedure common in projection-based embedding to obtain the Hartree–Fock orbitals for the quantum cores but rely directly on the initial Kohn–Sham orbitals. This not only simplifies our embedding approach significantly from a technical perspective but also allows us to use DFT orbitals for the correlated wave function region, which are often considered superior to Hartree–Fock orbitals.^33–35^

Effectively, our embedding approach is only a reformulation of (Huzinaga-type) projection-based embedding^19^ with multiple orbital sets that can be described by a correlated wave function method, i.e., the total Kohn–Sham energy was decomposed into energy contributions from individual orbital sets, which can then be used in different electronic structure models. As is typical for projection-based embedding, the orbitals of the LL region are kept frozen after partitioning into orbital sets and during the correlation treatment of the orbital sets assigned to quantum cores.

### 2.4 Transfer Machine Learning of Second-Level Embedding Energies

The second key component of our approach is a transfer learning step which connects the accurate quantum-core energies with the global potential energy surface represented in an ML/MM model.

In a brute-force approach (that is, without transfer learning), the structure−energy relation of the reference method would be directly learned by an ML potential.^36–40^ The ML potential training is based on representative structures and their total energies and gradients. However, calculating energies and gradients with multilevel embedding approaches requires high computational effort. In some cases, an implementation for the gradients may be unavailable. Therefore, training the QM/QM/MM energies *E*_QM/QM/MM_ from scratch can be challenging. Hence, we start here from a pretrained (system-focused) ML potential, such as the one obtained in our previous work.^18^ Typically, many more QM/MM than QM/QM/MM data points can be generated. These data points have already been generated to learn the QM/MM potential energy surface *E*_QM/MM_ in our previous work.

A refinement step will now be necessary to incorporate higher accuracy quantum data. Only the energy difference between QM/QM/MM and QM/MM Δ*E*_QM/QM/MM–QM/MM_ needs to be learned from the QM/QM/MM data

12 $$E_{\mathrm{QM}/\mathrm{QM}/\mathrm{MM}}=E_{\mathrm{QM}/\mathrm{MM}}+\Delta E_{\mathrm{QM}/\mathrm{QM}/\mathrm{MM}\text{−}\mathrm{QM}/\mathrm{MM}}$$

This correction is typically smoother than the full QM/QM/MM potential energy surface. Hence, fewer data points are required for sufficient sampling. The correction can be learned by a separate ML potential in a so-called Δ-learning approach.^41–43^ However, the inference requires then the evaluation of two MLPs. Training such a second MLP from scratch will again be difficult (for the same reason as highlighted before for the base MLP: the number of QM/QM/MM data points is small and/or no gradients are available for QM/QM/MM). Therefore, we apply transfer learning,^44–48^ where an MLP is first trained on QM/MM energies and gradients. Subsequently, some weight parameters of this MLP are fine-tuned to transfer its knowledge to the prediction of QM/QM/MM energies.

### 2.5 Practical Realization of Multilevel Learning

In the MLP approach toward a QM/MM potential energy surface described in ref 18, the MM energies *E*_MM_ and *E*_MM_^int^ are not learned since they can be calculated efficiently with the MM force field. We also separated in that approach electrostatic interactions between mixed QM–MM atom pairs from the energy to be learned. In ref 18, we calculated the total QM/MM energy *E*_QM/MM_ as

13 $$E_{\mathrm{QM}/\mathrm{MM}}=E_{\mathrm{ML}}^{\mathrm{QM}/\mathrm{MM}}+E_{\mathrm{MM}}+E_{\mathrm{LJ}}^{\mathrm{int}}+\sum_{I\in Q}\sum_{A\in E}\frac{q_{I}q_{A}}{\left|R_{I}-R_{A}\right|}+\sum_{m\in \mathrm{elements}}n_{m}E_{\mathrm{QM}}^{\mathrm{atomic},m}$$

The atomic charges *q*_*I*_ at positions ***R***_*I*_ of the QM atoms *I* and the charges *q*_*A*_ at positions ***R***_*A*_ of MM atoms *A* are taken from the MM force field in this work. *E*_QM_^atomic,*m*^ denotes the energy of a neutral atom of element *m* in its lowest energy spin state calculated with the QM method in the QM/MM approach. The number of atoms of element *m* in the QM region is denoted by *n*_*m*_. The atomic contributions to the QM energy *E*_QM_^atomic,*m*^ are separated because they represent a large part of the total QM energy. Therefore, separating them reduces the absolute value that must be learned, which simplifies training. Therefore, the ML potential energy *E*_ML_^QM/MM^ represents only the QM energy (*E*_QM_, without atomic contributions) and the difference in the electrostatic QM–MM interaction between electrostatic QM/MM embedding (*E*_elec_^int^) and the pure MM interaction

14 $$E_{\mathrm{ML}}^{\mathrm{QM}/\mathrm{MM}}=E_{\mathrm{QM}}+E_{\mathrm{elec}}^{\mathrm{int}}-\sum_{I\in Q}\sum_{A\in E}\frac{q_{I}q_{A}}{\left|R_{I}-R_{A}\right|}$$

In transfer learning, the MLP pretrained on the QM/MM data is fine-tuned on the energy *E*_ML_^QM/MM^ + Δ*E*_ML_^QM/QM/MM–QM/MM^. Since the QM/MM and QM/QM/MM potential energy surfaces can be shifted, the mean shift from QM/MM energies (without atomic contributions) to QM/QM/MM energies in the training data is handled separately

15 $$E_{\mathrm{QM}/\mathrm{QM}/\mathrm{MM}}=E_{\mathrm{ML}}^{\mathrm{QM}/\mathrm{MM}}+\Delta E_{\mathrm{ML}}^{\mathrm{QM}/\mathrm{QM}/\mathrm{MM}\text{−}\mathrm{QM}/\mathrm{MM}}+E_{\mathrm{MM}}+E_{\mathrm{MM}}^{\mathrm{int}}+\sum_{I\in Q}\sum_{A\in E}\frac{q_{I}q_{A}}{\left|R_{I}-R_{A}\right|}+\sum_{m\in \mathrm{elements}}n_{m}\left(E_{\mathrm{QM}}^{\mathrm{atomic},m}+{\overset{®}{\Delta E}}_{\mathrm{QM}/\mathrm{QM}/\mathrm{MM}\text{−}\mathrm{QM}/\mathrm{MM}}^{m}\right)$$

The shifts ${\overset{®}{\Delta E}}_{\mathrm{QM}/\mathrm{QM}/\mathrm{MM}\text{−}\mathrm{QM}/\mathrm{MM}}^{m}$ can be obtained in an element-dependent form by a least-squares fit of the differences in the QM/MM and QM/QM/MM training energies with respect to the stoichiometries.

We note that the specific choice for the shifts is not crucial for the whole training process. It eases the transfer learning, but any deficiencies of the shift (even a complete lack of the shift) can be compensated by the training process in the long run.

In conclusion, Δ*E*_ML_^QM/QM/MM–QM/MM^ represents only relative energy differences between QM/MM and QM/QM/MM approaches, centered around zero. Consequently, the MLP modifications during the training of the QM/QM/MM data should be as small as possible, enabling a sufficient representation by rather small amounts of data. For a leaner notation in the following, we introduce the shorthand notation *E*_ML_ = *E*_ML_^QM/MM^ + Δ*E*_ML_^QM/QM/MM–QM/MM^.

Optimizing the weight parameters only on the QM/QM/MM corrections can still lead to a loss of previous QM/MM information about the general shape of the potential energy surface. To mitigate this loss, a fraction of the MLP weight parameters are not modified. For the other weights, the state of the CoRe optimizer^49,50^ at the last training step of the QM/MM MLP is reloaded and continued, which includes learning rates individually adapted for each weight.

## 3 Computational Details

### 3.1 Multilevel Embedding

For the QM/QM/MM embedding, the initial DFT calculation for the full QM region applied the Perdew, Burke, and Ernzerhof exchange–correlation functional PBE,^51^ the D3 dispersion correction^52^ with Becke–Johnson damping (BJ),^53^ and the def2-TZVP basis set.^54^ The occupied core and valence orbitals were then localized separately, following the intrinsic bond orbital approach,^29^ and partitioned into orbital sets. Occupied orbitals were assigned to the environment if their Mulliken population exceeded 0.4 on the environment atoms. Otherwise, they were assigned to the quantum core on which they had the largest population. The electronic structure of the quantum cores was described by domain-based local pair natural orbital coupled cluster with singles, doubles, and semicanonical perturbative triples excitations [DLPNO-CCSD­(T_0_)] ^55,56^ using “NormalPNO” thresholds.^57^ Since the quantum cores were constructed from the Kohn–Sham DFT orbitals, the same basis functions were used for the cores. The shift ϵ_shift_ was chosen to be 1.0 au. All QM calculations were carried out with the Serenity program.^58–60^

The QM calculations were embedded in an MM environment by electrostatic embedding, the solvent, and the counterions. The parameters for the MM environment were taken from the Amber99SB-ILDN^61^ force field and TIP3P water model.^62^ The General Amber Force Field (version 2.11)^63,64^ provided the Lennard-Jones parameters for the noncovalent interaction between ligand atoms and the MM environment. The program Swoose^65,66^ calculated all force field terms.

The quantum cores were chosen according to the discussion in Section 2.2 and are illustrated in Figure 2. The first quantum core [blue in Figure 2(a)] consisted of the benzothiophene moiety of the ligand. The second quantum core [red in Figure 2(b)] consisted of the ligand’s mesityl chloride moiety (excluding the methyl groups). We calculated the QM/QM/MM energies for all structures of MCL1–19G and 19G that showed a QM/MM energy within 150 kJ mol^–1^ of the median of the respective QM/MM energy distribution.

We will refer to energies calculated with our QM/QM/MM approach as *E*_QM/QM/MM_, to energies calculated with the QM/MM (PBE-D3­(BJ)/def2-SVP-in-Amber) approach in ref 18 as *E*_QM/MM_, and to energies obtained from the initial DFT calculation (PBE-D3­(BJ)/def2-TZVP-in-Amber) for the full QM region during the embedding procedure as *E*_QM2/MM_.

### 3.2 Transfer Learning

The MLP was based on an ensemble of high-dimensional neural network potentials (HDNNPs).^36,67^ HDNNPs employ element-embracing atom-centered symmetry functions (eeACSFs) as their input features.^49^ As the QM/QM/MM MLP was trained based on the QM/MM MLP of ref 18, the eeACSF parameters and neural network architectures were chosen as in that reference. Furthermore, we continued to apply an ensemble size of 10 and an uncertainty scaling factor of *c* = 2.^49^

The set of QM/QM/MM reference energies was split randomly into 90% training and 10% test data for each ensemble member individually unless stated otherwise. Transfer learning employed the CoRe optimizer^49,50^ (version 1.1.0)^68^ to minimize the difference in energy predictions and respective training data. The hyperparameters of the CoRe optimizer were taken from ref 18. The optimizer state of the last training step in learning the QM/MM potential energy surface was loaded, and the training was continued. In each of the 300 training steps, all training energies were exploited. The weight parameters for the standardization of the input features^49^ and those of the first hidden layer of the neural networks were not varied in transfer learning.

Transfer learning and inference of the MLPs were carried out with a development version of the lMLP software applied in ref 18. The program lMLP is available on GitHub, PyPI, and included in the Zenodo archive.^66^

As training data for the transfer learning of the QM/QM/MM-based ML­(II) model, we employed all structures for which QM/QM/MM energies were calculated, as discussed in Section 3.1. In total, there were 8417 structures for the protein–ligand complex and 5285 structures for the solvated ligand. For the study of the effect of exploiting also energy gradients (in addition to the energies) on the transfer learning in Section 4.3, we relied on 10^4^ structures from the MM trajectories for the solvated ligand and the protein–ligand complexes.

### 3.3 End-State Correction Simulations

The nonequilibrium (NEQ) switching simulations for the end-state corrections in the thermodynamic cycle were run as described in ref 18. In this protocol, 150 structures were randomly selected from the MM end states. For these structures, the force field was switched from MM to ML­(II)/MM over 10 ps, using an NPT ensemble and the same conditions employed in the MM simulations. After the forward switch, we resampled the structures to represent best the equilibrium distribution of the ML­(II)/MM potential energy surface, propagated the structures for another 10 ps, and then switched the potential energy surface again from ML­(II)/MM to MM. This NEQ switching procedure was repeated six times for the protein–ligand complex and the solvated ligand to estimate the uncertainty from the sampling during NEQ switching.

We estimate the uncertainty δ*G*_bind_^ML(II)/MM^ for the binding free energy with the error from the underlying MM calculation and the sampling error from the NEQ simulations and Multistate Bennett acceptance ratio (MBAR)^69^ as

16 $$\delta G_{\mathrm{bind}}^{\mathrm{ML}\left(\mathrm{II}\right)/\mathrm{MM}}=\sqrt{{\left(\delta G_{L+P}^{\mathrm{MBAR}}\right)}^{2}+{\left(\delta G_{L+S}^{\mathrm{MBAR}}\right)}^{2}+{\left(2\sigma_{\mathrm{bind}}^{\mathrm{ML}\left(\mathrm{II}\right)/\mathrm{MM}}\right)}^{2}}$$

Here, δ*G*_L+S_^MBAR^ and δ*G*_L+P_^MBAR^ denote the MBAR error estimates for the solvated ligand and the protein–ligand complex, respectively, and σ_bind_^ML(II)/MM^ denotes the standard deviation of the 36 binding free energy estimations (6 × 6 for each end state).

## 4 Results for Binding Free Energies from Hierarchical Quantum Hybrid Models

### 4.1 Energy Distributions in Quantum Hybrid Models

Since we aim at nonequilibrium switching simulations to approximate the free energy difference between the QM/MM and the QM/QM/MM potential energy surfaces, we initially investigated the difference in relative energies between both approaches. The initial QM/MM model and our QM/QM/MM approach differ in two aspects. (i) We employed the def2-TZVP basis set in the QM/QM/MM calculations, but only the smaller def2-SVP basis set in the initial QM/MM calculation. (ii) The QM/QM/MM approach described two quantum cores with DLPNO-CCSD­(T_0_).

To disentangle the effect of the basis set and the QM/QM embedding, we plotted the total energy difference Δ*E*_tot_ = *E*_QM/QM/MM_ – *E*_QM/MM_, the difference in the energies caused by the basis set Δ*E*_Basis_ = *E*_QM2/MM_ – *E*_QM/MM_, and the difference in the energies caused by the QM/QM embedding Δ*E*_Emb_ = *E*_QM/QM/MM_ – *E*_QM2/MM_ in Figure 3.

**Figure 3:**
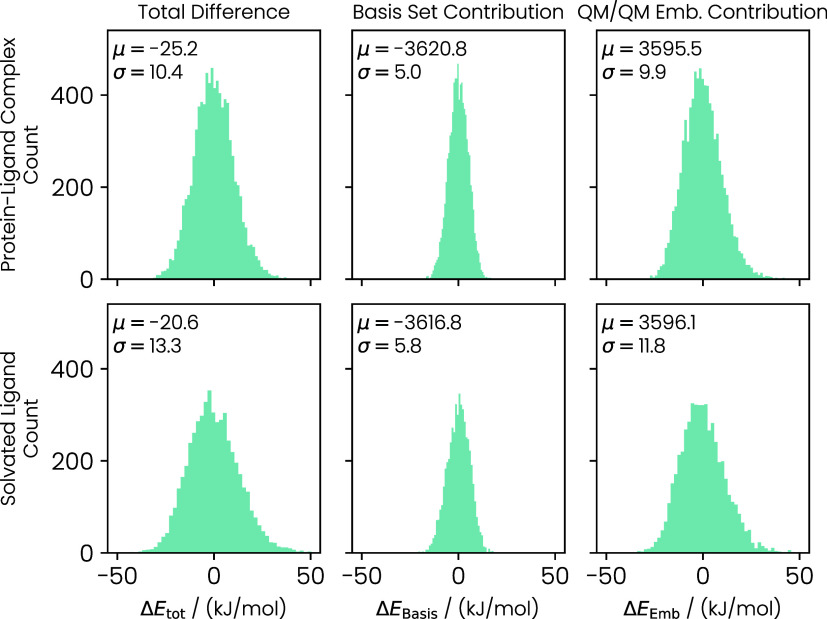
Distributions of the energy differences of Δ*E*_tot_, Δ*E*_Basis_, and Δ*E*_Emb_ for the protein–ligand complex and the ligand in solution. The distributions are shifted by their mean for clarity. The standard deviations (σ) and means (μ) are given in kJ mol^–1^.

The distributions for the energy differences for both end states (protein–ligand complex and solvated ligand) are symmetric and almost of Gaussian shape. Overall, the distribution for the total energy differences Δ*E*_tot_ are relatively narrow with standard deviations of 10.4 kJ mol^–1^ and 13.3 kJ mol^–1^ for the protein–ligand complex and the solvated ligand, respectively. The standard deviations for Δ*E*_Emb_ are 9.9 and 11.8 kJ mol^–1^ for the respective end states, which accounts for most of the variance in Δ*E*_tot_. By contrast, increasing the basis set size from def2-SVP to def2-TZVP results only in a variance of 5.0 and 5.8 kJ mol^–1^ for the protein–ligand complex and the solvated ligand, respectively. Since we need to calculate the energy difference between both end states, the mean differences of the energy distributions provide a first hint for the effect on the final free energy differences. Here, the means of Δ*E*_tot_ for the protein–ligand complex and solvated ligand differ by −4.6 kJ mol^–1^, suggesting that the QM/QM embedding stabilizes the bound state. The majority of this mean shift (−4.0 kJ mol^–1^) is caused by the improved basis set, while the remaining −0.6 kJ mol^–1^ can be traced back to the QM/QM embedding.

### 4.2 Accuracy of the Machine Learning Potential

For both systems, the protein–ligand complex and the solvated ligand, a separate MLP is trained to achieve the best accuracy−cost ratio for inference. Transfer learning is based on 8417 reference conformers for the protein–ligand complex and 5285 for the solvated ligand, while each reference conformer contains *N*_*Q*_ = 44 QM atoms. The ranges of the target reference QM/QM/MM energies per QM atom *E*_ML_^ref^ *N*_*Q*_^–1^ are 8.649 and 8.756 kJ mol^–1^, respectively, while the standard deviations are 0.845 and 0.857 kJ mol^–1^.

After training, root-mean-square energy errors (RMSEs) are an order of magnitude smaller than the energy ranges and considerably smaller than their standard deviations, confirming the high fidelity (Table 1). Training and test data show similar errors indicating the absence of overfitting. Ensembling of ten HDNNPs in an MLP reduces the RMSE values compared to the individual predictions, especially for the solvated ligand.

**Table 1:** RMSEs of Target QM/QM/MM Energies *E*_ML_ for HDNNPs Prior to Ensembling and of HDNNP Ensembles^a^

RMSEs before ensembling	MCL1–19G	19G
*E*_ML_^train^*N*_*Q*_^–1^ (kJ mol^–1^)	0.159 ± 0.004	0.218 ± 0.005
*E*_ML_^test^*N*_*Q*_^–1^ (kJ mol^–1^)	0.169 ± 0.007	0.243 ± 0.009
**ensemble RMSEs**		
${\mathrm{E̅}}_{ML}N_{Q}^{-1}\;\left(kJ\;{mol}^{-1}\right)$	0.150	0.205

a The mean and standard deviations of ten individual HDNNPs are provided for training and test data. The HDNNP ensemble was applied to all data.

The QM/QM/MM MLP RMSEs are 5 and 14% lower than those of the base QM/MM MLPs of the protein–ligand complex and for the solvated ligand, respectively. Whereas the standard deviations of the training data for the ML­(I) potential were 3.6 kJ mol^–1^ for the protein–ligand complex and 5.9 kJ mol^–1^ for the solvated ligand, the standard deviations for the training data used here turned out to be 0.8 and 0.9 kJ mol^–1^, respectively. Therefore, the increased accuracy can be attributed to the narrower energy distribution of the training data, a consequence of the restriction to structures within 150 kJ mol^–1^ of the median energy.

The error distribution in Figure 4 shows that the errors are well centered around zero and that the majority of data points show absolute errors smaller than 0.5 kJ mol^–1^ per QM atom. The training process in Figure 5 highlights the smooth convergence of the transfer learning approach as well as that of the CoRe optimizer.

**Figure 4:**
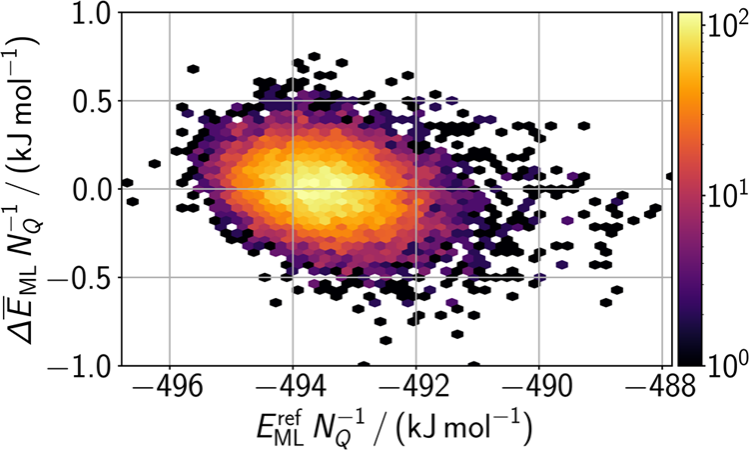
Difference in the ensemble prediction of target QM/QM/MM energies $\Delta {\mathrm{E̅}}_{\mathrm{ML}}$ from the reference data *E*_ML_^ref^ as a function of the reference data *E*_ML_^ref^ for both systems, MCL1–19G and 19G, normalized by the number of QM atoms *N*_*Q*_. Color in this hexagonal binning plot visualizes the number of data points in a hexagon. Three outlier data points are outside the shown error range.

**Figure 5:**
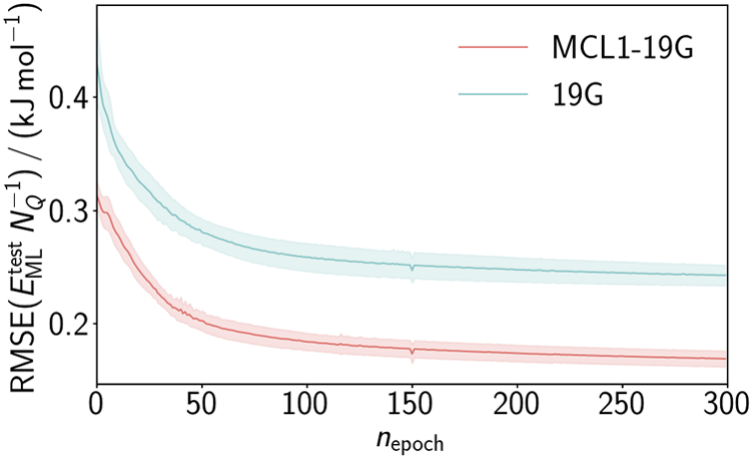
Transfer learning progress of RMSE­(*E*_ML_^test^ *N*_*Q*_^–1^) of both systems, MCL1–19G and 19G, as a function of the training epoch *n*_epoch_. The solid line represents the mean of ten individual HDNNPs and the shaded area shows the standard deviation.

### 4.3 Effect of Energy Gradients and Amount of Training Data on Transfer Learning

Atomic forces, i.e., negative energy gradients, are not available in our current implementation of the QM/QM/MM energies. However, training on forces may significantly improve the learning process because they provide information about the local shape of the potential energy surface. To probe the extent to which forces can affect the resulting accuracy of a transfer learned MLP, we constructed an embedding approach where the QM region is described by an MM force field. In this way, we can train a base model using 10^4^ snapshots from the MM trajectory on MM/MM energies and apply transfer learning to the QM/MM potential energy surface, while in both cases, energy gradients are available. In addition, we can analyze the quality of the resulting force predictions for transfer learning, which utilizes only energies. Furthermore, direct learning of the QM/MM data also allows us to obtain a reference for the resulting accuracy.

Transfer learning only on energies yields similar RMSEs for the energies *E* ^QM/MM^ as transfer learning on energies and forces (Table 2). However, the resulting RMSE for the atomic force components of the QM atoms *F*_α,*n*(*Q*)_^QM/MM^ is about twice as high for training only on energies compared to training on energies and forces. A higher force RMSE for the former learning case is expected as much less information about the potential energy surface is available. Each of the 9·10^3^ training conformers provides only one energy value, while 3 · 44 force components of QM atoms are available. For the RMSE of the atomic force component of the MM atoms represented by the MLP *F*_α,*n*(*E*′)_^QM/MM^, the difference between the learning cases is not as large. However, the absolute values of these force errors are, in general, 1 order of magnitude smaller than those of *F*_α,*n*(*Q*)_^QM/MM^, which leads to less emphasis on *F*_α,*n*(*E*′)_^QM/MM^ during training.

**Table 2:** RMSEs of QM/MM Energies *E* ^QM/MM^ and QM/MM Atomic Force Components of QM Atoms *F*_α,*n*(*Q*)_^QM/MM^ and MM Atoms Represented by the MLP *F*_α,*n*(*E*′)_^QM/MM^ for HDNNP Ensembles Evaluated on All Data, i.e., Training and Test Data^a^

		RMSE	RMSE	RMSE
learning	system	${\mathrm{E̅}}^{QM/MM}N_{Q}^{-1}$ (kJ mol^–1^)	${\mathrm{F̅}}_{\alpha ,n\left(Q\right)}^{\mathrm{QM}/\mathrm{MM}}$ (kJ mol^–1^ Å^–1^)	${\mathrm{F̅}}_{\alpha ,n\left(E\prime \right)}^{\mathrm{QM}/\mathrm{MM}}$ (kJ mol^–1^ Å^–1^)
transfer *E*	MCL1–19G	0.150	22.1	1.01
	19G	0.195	23.7	1.81
transfer *E* and *F*	MCL1–19G	0.145	11.3	0.94
	19G	0.212	12.8	1.73
direct	MCL1–19G	0.117	9.0	0.81
	19G	0.161	10.1	1.48

a The HDNNP ensembles were obtained by training on pure MM data and transfer learning to QM/MM data, while either only energies or energies and forces were exploited in transfer learning. Direct training on QM/MM data is given as a reference. The underlying 10^4^ reference conformers were obtained from MM sampling during AFE simulations.

Comparing the results of transfer learning to direct learning shows that the energy RMSE increases by around 26% (Table 2). Here, the same number of reference conformers are employed, but still the flexibility of the ML potential parameters and the number of updates during transfer learning are significantly lower than for direct learning. For *F*_α,*n*(*Q*)_^QM/MM^, transfer learning energies and forces also yields an increase of the RMSE of about 26% compared to direct learning. The increase is about 16% for *F*_α,*n*(*E*′)_^QM/MM^. Still, the RMSEs are decent for the following Alchemical Free Energy (AFE) simulations, even if the transfer learning is only based on energies since the RMSEs are in a similar range as those of previously employed HDNNPs in simulations on pure QM potential energy surfaces.^70,71^

To understand how many energy values for training are required in transfer learning for the representation of the conformation space sampled by MM in AFE simulations, the training data fraction *p*_train_ was varied. We observe that reducing the training fraction of the 10^4^ reference conformers from 0.9 to 0.3 increases the resulting test RMSEs of energies and forces only little (Figure 6(a–c)). Decreasing the number of training conformers to 10^3^ leads to increases in the *E* ^QM/MM^ and *F*_α,*n*(*Q*)_^QM/MM^ RMSEs of about 12% compared to utilizing 9 · 10^3^ conformers. If the number is further decreased to 0.5·10^3^, the RMSEs will increase by about 25%. Consequently, the MLP accuracy is still fine, even if only about a tenth of the conformers and only energies are employed. The smoothness of the transfer learned potential energy surface is probed in the AFE simulations presented in the next section. These simulations demonstrate that the forces of the QM atoms, which are predicted by an MLP obtained in transfer learning only on energies, lead to reasonable conformational ensembles in the simulations.

**Figure 6:**
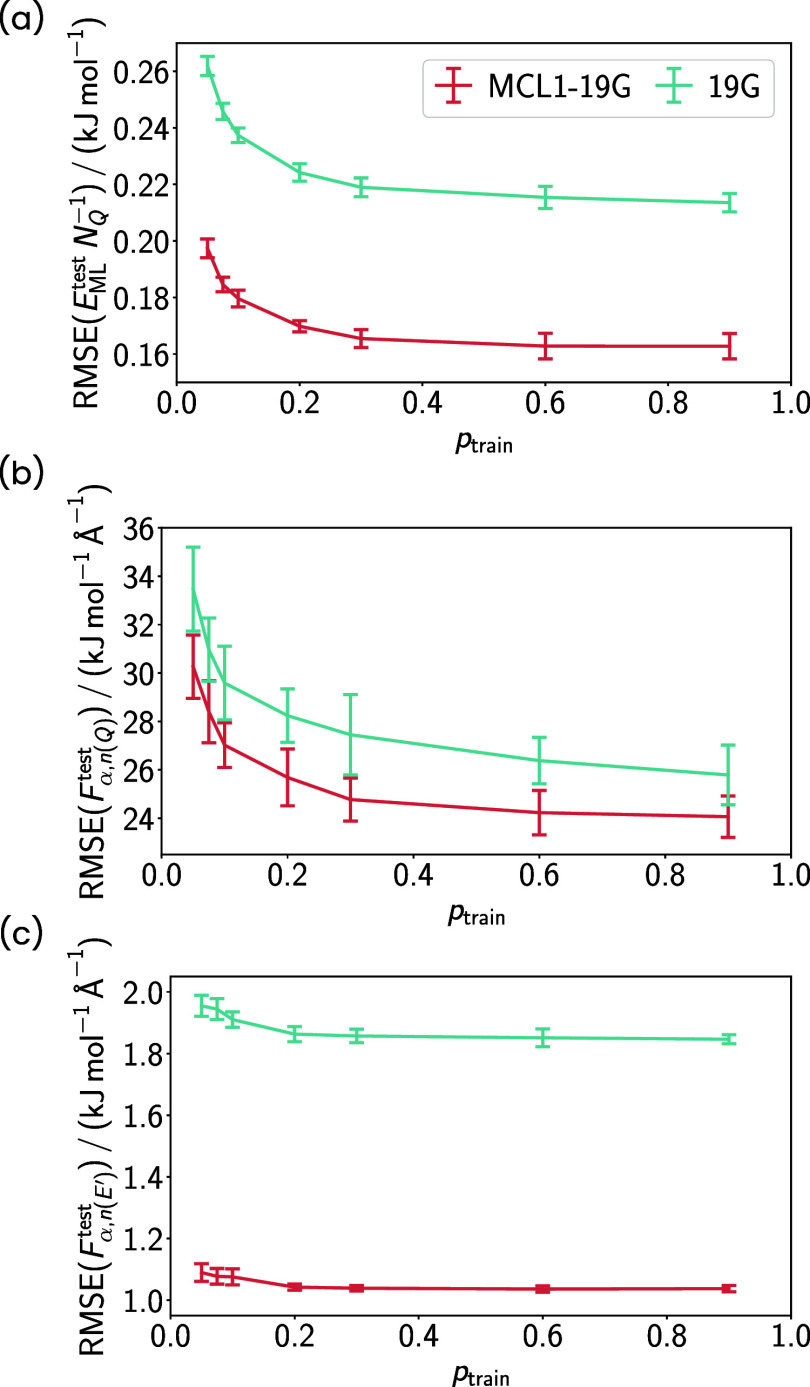
Test set RMSEs of (a) QM/MM energies *E*_ML_^test^ and (b) QM/MM atomic force components of QM atoms *F*_α,*n*(*Q*)_^test^ and (c) MM atoms represented by the ML potential *F*_α,*n*(*E*′)_^test^ as a function of the training fraction *p*_train_. The lines represent the mean of ten individual HDNNPs predictions, i.e., ensembling is not applied, and the error bars show the standard deviations. The HDNNPs were obtained by training on pure MM data and transfer learning to different amounts of QM/MM data, while only energies were utilized in transfer learning. The underlying 10^4^ reference conformers were obtained from MM sampling during AFE simulations.

### 4.4 Free Energy Corrections from Non-Equilibrium Switching

The work distributions for the first out of six NEQ switching simulations are shown in Figure 7 for the protein–ligand complex and the solvated ligand. For both end states, we observe two distinct peaks in the work distribution. In ref 18, these peaks have already been related to the preference of the carboxylic acid to form hydrogen bonds to sulfur or the solvent. On the MM potential energy surface, the ligand forms a hydrogen bond with a neighboring sulfur atom for some conformations. These hydrogen bonds are fully replaced by hydrogen bonds to solvent molecules after switching to the MLP. Therefore, switches starting from MM conformations with hydrogen bonds to a solvent molecule require less work. Furthermore, the system does not switch back to hydrogen bonds with sulfur during the short time of the backward switch.

**Figure 7:**
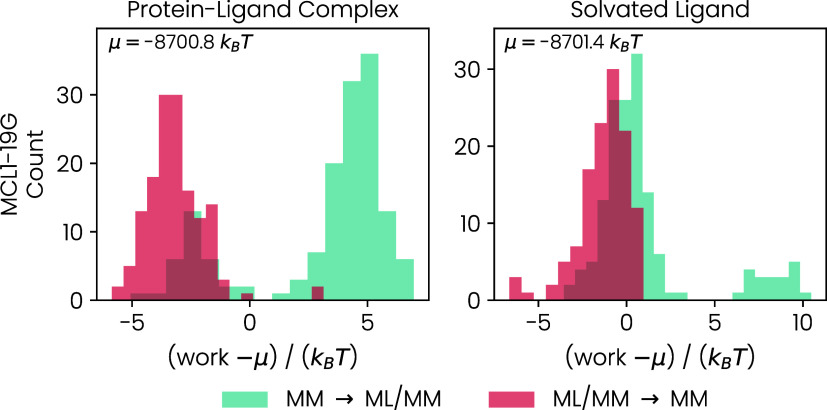
Work distributions from NEQ switching simulations. The results for the protein–ligand complex are shown in the left plot and those for the solvated ligand in the right plot.

We estimated the binding free energy of the ligand 19G to the protein MCL1 as Δ*G*_bind_^ML(I)/MM^ = −35.3 kJ mol^–1^ ± 1.8 kJ mol^–1^,^18^ underestimating the binding by 1.9 kJ mol^–1^ compared to the experimental estimate (−37.3 kJ mol^–1^ ± 0.1 kJ mol^–1^ ^20^). After generating MLP­(II) through transfer learning, we carried out six NEQ switching simulations for the protein–ligand complex and the solvated ligand. We calculated the mean of the resulting 36 binding free energies ⟨Δ*G*_bind_^ML(II)/MM^⟩ = −37.2 kJ mol^–1^ ± 1.0 kJ mol^–1^, which closely matches the experimental estimate. However, we note here that the protonation state of the ligand is unclear since its carboxylic acid group is likely deprotonated in solution. Therefore, this close agreement with the experimental estimate may have benefited from error cancellation. We chose the neutral ligand to avoid charge compensation during the initial MM AFE calculation.

## 5 Conclusions

In this work, we have developed a three-level QM/QM/MM approach that can be exploited to refine QM/MM potential energy surfaces by allowing for highly accurate quantum chemical calculations in quantum cores, which are embedded into the large QM region of a QM/MM model. In this way, a higher accuracy for substructures can be fed into a machine learning representation of the potential energy surface. This is brought about by projection-based embedding and transfer learning of a machine learning potential. As a result, the large QM region of the initial QM/MM model, typically described by DFT or any other semiempirical approach, can be locally improved by correlated coupled cluster calculations as demonstrated in this work. Although the combination of semiempirical methods^72^ with projection-based embedding has not been shown yet, employing such methods should be feasible as long as a sufficiently accurate dual-basis approach^16^ can be exploited. Depending on the nature of the electronic structure of the quantum cores, also multiconfigurational approaches are applicable and even quantum computation^73–76^ can step in as a source of high-accuracy quantum energies, as we will demonstrate in a forthcoming paper.^77^

We demonstrated the capabilities of this approach for binding free energies of protein−drug complexes, typically investigated with MM-only approaches. Our approach relies on an end-state correction through nonequilibrium switching of MM-based alchemical free energy simulations. The nonequilibrium switching calculations are made feasible by training MLPs on QM/MM energies and forces for the protein–ligand complex and the solvated ligand. These QM/MM-based MLPs were then improved through transfer learning with QM/QM/MM energies.

We validated our approach using the well-studied protein–ligand complex of the inhibitor 19G binding to myeloid cell leukemia 1. The QM/QM/MM embedding provided a locally corrected description of the electronic structure, which had a small but non-negligible effect on the relative energy distributions for the system under study. The QM/QM/MM-based MLP provided a binding free energy for MCL1–19G in close agreement with the experimental estimate, improving on our previous single-level embedding QM/MM-based approach.^18^ Although in this particular case, the electronic structure in the quantum cores has been improved mostly by introducing a larger atomic orbital basis set (and to a much smaller extent by the more accurate electronic structure model provided by coupled cluster theory), both contribute to the overall improvement within the quantum cores. Hence, the methodology proposed in this paper demonstrates that this eventually improves on the free energy calculation. Moreover, it must be emphasized that the electronic structure in the quantum cores can be multiconfigurational and/or open-shell, so that far larger effects of a better electronic structure model will be seen, as we shall demonstrate in a forthcoming paper.

However, one may also adopt a different point of view and take our results as an objective way to confirm that a DFT-embedding approach actually works sufficiently well in a QM/MM hybrid model. Hence, our nested embedding approach allows one to probe rigorously the accuracy of a lower-accuracy model at the QM/MM embedding level.

Furthermore, we investigated the importance of energy derivatives for learning the potential energy surface with transfer learning from QM/QM/MM data. To this end, we trained a machine learning potential to predict the MM energies and forces. We then compared the performance of transfer learning from this MM-ML potential to QM/MM using only energies and using energies and forces. Training with only energies increased the errors for the forces, suggesting that significantly more data points are required to reach a similar accuracy as the ML potential trained with energies and forces. This demonstrates that energy derivatives are key for the efficient training of MLPs and provide additional incentive to develop analytical expressions for energy derivatives of highly accurate electronic structure methods, such as DLPNO-CCSD­(T_0_), within embedding frameworks, which are, however, currently unavailable.

We also want to note that the specific selection of the quantum cores may have a nonnegligible effect on the overall description. Very small embedded regions in Huzinaga-based embedding can decrease the accuracy of relative energies compared to a full DFT description since error cancellation intrinsic to DFT is lost.^32^ However, these problems can be addressed by more accurate exchange−correlation functionals, such as double hybrid functionals, or by increasing the size of the quantum cores (for instance, through automated selection procedures).^26^

In future work, we will investigate active learning strategies for transfer learning to lower the computational cost of our approach. Such an active learning strategy aims to identify the structures for which the QM/QM/MM and QM/MM potential energy surfaces are significantly different, and a QM/QM/MM calculation is required to correct the PES locally.

## Data Availability

The transfer learned machine learning potentials, the databases containing all QM/QM/MM and QM/MM energies, and the final work distributions for all NEQ simulations are available on Zenodo.^66^
